# Organ specificity and transcriptional control of metabolic routes revealed by expression QTL profiling of source--sink tissues in a segregating potato population

**DOI:** 10.1186/1471-2229-12-17

**Published:** 2012-02-07

**Authors:** Bjorn Kloosterman, AM Anithakumari, Pierre-Yves Chibon, Marian Oortwijn, Gerard C van der Linden, Richard GF Visser, Christian WB Bachem

**Affiliations:** 1 Wageningen UR Plant Breeding, Wageningen University and Research Center, PO Box 386, 6700 AJ Wageningen, the Netherlands; 2 Graduate School Experimental Plant Sciences, Wageningen, The Netherlands; 3 Centre for BioSystems Genomics, P.O. Box 98, 6700 AA Wageningen, The Netherlands; 4 KeyGene N.V., P.O. Box 216, 6700 AE Wageningen, The Netherlands

## Abstract

**Background:**

With the completion of genome sequences belonging to some of the major crop plants, new challenges arise to utilize this data for crop improvement and increased food security. The field of genetical genomics has the potential to identify genes displaying heritable differential expression associated to important phenotypic traits. Here we describe the identification of expression QTLs (eQTLs) in two different potato tissues of a segregating potato population and query the potato genome sequence to differentiate between cis- and trans-acting eQTLs in relation to gene subfunctionalization.

**Results:**

Leaf and tuber samples were analysed and screened for the presence of conserved and tissue dependent eQTLs. Expression QTLs present in both tissues are predominantly cis-acting whilst for tissue specific QTLs, the percentage of trans-acting QTLs increases. Tissue dependent eQTLs were assigned to functional classes and visualized in metabolic pathways. We identified a potential regulatory network on chromosome 10 involving genes crucial for maintaining circadian rhythms and controlling clock output genes. In addition, we show that the type of genetic material screened and sampling strategy applied, can have a high impact on the output of genetical genomics studies.

**Conclusions:**

Identification of tissue dependent regulatory networks based on mapped differential expression not only gives us insight in tissue dependent gene subfunctionalization but brings new insights into key biological processes and delivers targets for future haplotyping and genetic marker development.

## Background

The field of associative genomics or genetical genomics attempts to combine the heritability of generated ~ omics data with phenotypic variation through genetic marker associations [1]. The aimed outcome of a genetical genomics study is to define genomic regions that control the expression of single or multiple genes, metabolites and/or proteins (eQTLs, mQTLs and pQTLs, respectively). The amount of population wide ~ omics data for non-model species has increased dramatically over the last few years as costs for data generation have decreased and computational bottlenecks have been largely overcome. Successful application of genetical genomics has been demonstrated for a number of plant species including Arabidopsis, barley, wheat, eucalyptus and poplar [2-8]. In particular, studies with the model plant Arabidopsis have been crucial for the development of the concept of genetical genomics and have pushed software development forward to cope with the increasing amount of data generated by the different profiling platforms available [1,9]. Associations between ~ omics data, markers and phenotypes have highlighted the overall complexity of plant trait diversity and have led to the reconstruction of a number of regulatory networks including flowering [4], glucosinolates [10], flavonoids [11] and carotenoids [12]. The identification of eQTL hotspots may indicate the presence of major regulatory switches controlling the expression of many genes directly or indirectly [13]. The existence of eQTL hotspots was confirmed in a number of studies were an enrichment of gene functional categories was found for several of the identified regions [14,15]. However, network modelling still heavily relies on a-prior knowledge of the pathways targeted [16].

Despite the high potential of genetical genomics studies in plants, the association of differential gene expression or metabolite concentration with the phenotypic variation does not always disclose the responsible genetic factors, due to genetic linkage of additional genes with the genomic region. Identification of causative polymorphisms is important for the development of applicable genetic markers. For genetical genomics experiments, factors like population size, marker density, sampling strategy, environmental factors and screening platform used, greatly influence the outcome and the ability to detect small additive biological effects [reviewed in [17,18]]. The high costs for population-wide screening often limit genetical genomics experiments to a single tissue or developmental stage. Several studies indicate that the genetic architecture of gene expression is highly divergent between organs and/or developmental stages which will be reflected in eQTL tissue specificity [5,19-21]. In poplar it was found that less than one-third of genes with eQTLs have co-localizing eQTLs when comparing two different organs [5]. Gene duplication events and diversification through mutations, referred to as sub- and neofunctionalization, underlie gene expression differences and are hypothesized to be the driving forces for obtaining novel gene functions and consequently evolution of plant development [22,23].

Potato cultivars are highly heterozygous tetraploid outcrossing plants, which complicates genetic analysis aimed at understanding the molecular mechanisms underlying trait variation. Genetic studies therefore are often performed in diploid genotypes and populations. Precision breeding relies on the identification of causative polymorphisms in genes responsible for trait variation. Once a gene has been assigned a regulatory function for a targeted trait, the allelic diversity of the gene in tetraploid cultivars and wild type accessions can be determined and potentially exploited in marker assisted breeding [24].

The genome sequence of a dihaploid potato clone (DM) has recently been completed [25] and this will facilitate the rapid identification and cloning of genes associated to trait variation and thus haplotyping efforts. Genetical genomics experiments in potato opens up a new dimension with the availability of the genome sequence. With the physical as well as the genetic map location of many genes known, QTL intervals can be more easily screened for candidate genes. Here we present the first genetical genomics study in potato with eQTL analysis in both leaves and tuber. A comparison between leaf and tuber tissues reveals conservation as well as tissue dependent variation of gene expression. Gene interactions and potential regulatory networks are identified and discussed.

## Results and discussion

### QTL analysis and distribution

Expression profiles of leaf and tuber RNA extracts were obtained from two independent experiments. For tuber profiling, each sample was assayed twice in a two-colour dye experiment, allowing cross-validation of the observed variation and subsequent filtering based on correlation scores (Methods). The total number of array features exhibiting significant expression that could be used for QTL mapping was higher for leaf (22,193) than tuber samples (19,956). A wide overlap of expressed genes (19,590 features) was found between both organs, which was somewhat surprising considering their highly contrasting functions in terms of architecture and morphology, environmental exposure and developmental stage. However, the organs harvested consist of a multitude of different cell types thereby reducing the overall transcriptomic complexity and no distinction between basal or high gene expression was made. Similarly, the potato microarray is based on available EST sequences where temporal and spatially restricted transcripts have a lower change of being captured and thus not profiled when using the microarray.

QTL analysis was performed based on the R/qtl package that allows high-throughput QTL mapping in an outbreeding species using a single integrated genetic map (Methods). A total of 17,764 QTLs were identified as significant for leaf and tuber data combined, corrected for gene redundancy present on the array where possible (i.e. similar blast scores for independent array features against the potato genome gene prediction sequences v3.2).

**Figure 1:**
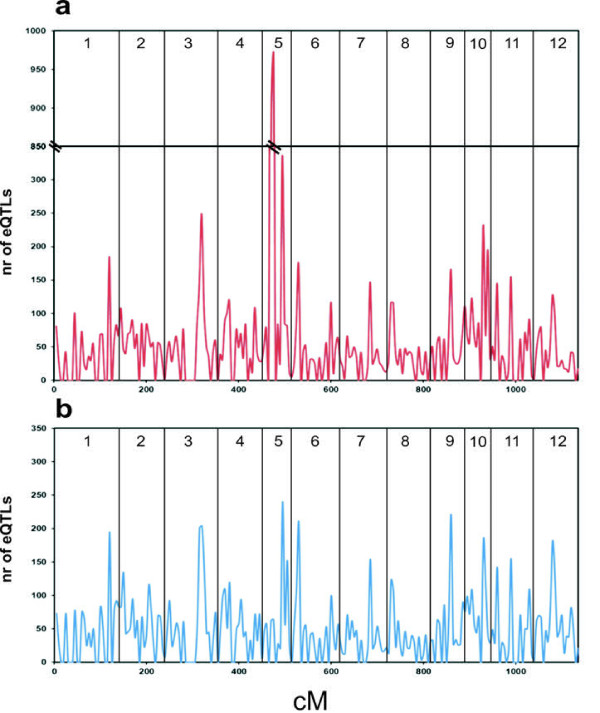
**Genome-wide eQTL distribution in potato**. Overview of genome-wide eQTL distribution within the C × E population over the 12 potato linkage groups for tuber (**a**) and leaf (**b**) gene expression data. The total number of significant eQTLs found within 5 cM intervals is plotted along the genetic map (cM).

The distribution of QTLs over the different linkage groups is presented as a sliding window graph using 5 cM stepping size for tuber and leaf (Figures 1a and 1b, respectively). The number of significant QTLs for each linkage group is provided in Additional file 1. A clear over-representation of the number of QTLs for tuber expression was detected on linkage group 5 with over 1800 QTLs mapped in just two neighbouring bins (20-30 cM). For leaf expression data, the eQTLs appear to be more evenly distributed over the genome, although several genetic intervals can be identified in which the number of QTLs is high (> 200 on chromosomes 3, 5, 6, and 9). When comparing leaf and tuber eQTL distribution overlapping regions with high eQTL numbers can be identified. These regions may indicate regions of high gene density or cover large physical sequence distances as a result of low recombination frequencies. Not all markers used in this study can be linked to the genomic scaffolds and not all scaffolds have been anchored onto the physical map, which needs to be resolved before answering these questions.

For the majority of features on the array (12,331; 70.1%), only a single QTL was detected while for 2,503 features (29.9%), two or more QTLs were found for both tissues combined (Table 1a). For two features on the array, a maximum of 5 QTLs were identified. One shows strong homology to a *tropinone reductase II *(TRII; EST *cSTB29M16TH*) [26] involved in the biosynthesis of tropane alkaloids which is known to be expressed in both below- and above-ground potato tissues. The second feature (BF_LBCHXXXX_0032H04_T3M.SCF) with 5 different QTLs has no significant homology to any protein present in the databases nor was its gene structure predicted in the current release of the potato genome annotation [25]. For both genes, four QTLs were detected in leaves and a fifth non-overlapping QTL in tubers. In a study from West et al. [2], the number of regions reported to control the expression of a gene in Arabidopsis ranges from 0 to 11 with an average of 2.34 transcript per gene. In a barley study [3], over a third of regulated genes had only one identified eQTL while the rest of the genes showed 2-6 eQTLs. In our dataset we found relative few genes that were controlled by more than 2 genomic regions (2503 features) with a calculated average of 1.19 QTL per gene. Estimations of the average number of genomic regions (QTLs) controlling the expression of a single gene varies depending on the algorithm used, the population type and size [3,27]. The number of eQTLs per gene identified in this study is likely to be an underestimation, as genes with small additive effects may remain undetected due to the relative small population size (n = 94) and marker density resulting in a high LOD threshold (4.35). Moreover, here we have targeted mature tubers and open leaves, to allow analysis of source--sink relationships, while more temporal and spatially restricted gene expression can be found during organ initiation and in young developing tissues.

**Table 1:** Overview of eQTL analysis results based on obtained population-wide gene expression profiles for potato tuber and leaf tissue

A						
Nr of QTLS per Feature*	**1**	**2**	**3**	**4**	**5**	**Total nr of QTLs**

**Nr of Features**	12331	2269	213	19	2	**17594**

**B**						

**QTL Expl. Var. %**	**> 10%**	**> 20%**	**> 30%**	**> 50%**	**> 70%**	**> 90%**

**Nr of QTLs Tuber**	12192	10974	7306	3930	1856	293

**Cis (%)**	7550 (61.9)	7243(66.0)	5699 (78.0)	3279 (83.4)	1515 (81.6)	248 (84.6)

**Trans (%)**	3629 (29.8)	2792(25.4)	935 (12.8)	269 (6.8)	127 (6.8)	10 (3.4)

**Unknown (%)**	1013 (8.3)	939 (8.6)	672 (9.2)	382 (9.7)	214(11.5)	35 (11.9)

**Nr of QTLs Leaf**	10745	9629	6644	3884	1897	388

**Cis (%)**	7574 (70.5)	7211 (74.9)	5517 (83.0)	3239 (83.4)	1552 (81.8)	297 (76.5)

**Trans (%)**	2180 (20.3)	1536 (16.0)	497 (7.5)	255 (6.6)	118 (6.2)	27 (7.0)

**Unknown (%)**	991 (9.2)	882 (9.2)	630 (9.5)	390 (10.0)	227 (12.0)	64 (16.5)

**C**						

**Tissue Comparison**	**Nr of eQTLs**	**Cis (%)**	**Trans (%)**	**Unknown (%)**		

**Leaf + Tuber eQTL**	5818	4929 (84.7)	401 (6.9)	488 (8.4)		

**Leaf Unique eQTL**	5126	2839 (55.4)	1784 (34.8)	503 (9.8)		

**Tuber unique eQTL**	6650	2882 (43.3)	3243 (48.8)	525 (7.9)		

Tissue dependent**	1713	530 (30.9)	977 (57.0)	285 (12.0)		

* Leaf + Tuber data

** eQTL both in leaf and tuber but non-overlapping

### Cis vs. Trans-acting eQTls

One of the most exciting outputs from large-scale eQTL studies in species with a full genome sequence available is the ability to distinguish cis- and trans-acting regulation of gene expression, which holds the potential to reveal regulatory networks. With the availability of the potato draft genome sequence we were able to distinguish cis- and trans-acting QTLs for most genes. EST based unigenes representing the features on the array were blasted against the genome scaffolds as described in Methods. Putative chromosome locations could be assigned to the vast majority of genes (92%). Genes that could not be assigned a chromosome location were either similar to multiple regions on the genome, had similarity scores below the threshold, or represented significant allelic diversity.

Identified QTLs on the same linkage group as their physical map position are identified as cis-acting while QTLs on different linkage groups are defined as trans-acting. Our results show that almost twice as many cis-eQTLs were identified in comparison to trans-acting eQTLs for leaf and tuber data (Table 1b; > 10% explained variance). This may indicate that in potato there is a strong preference for cis- over trans-acting transcriptional control, which is in contrast with observations made in other studies where trans-acting QTLs are generally overrepresented [2,4,5,28]. Although the low mapping resolution and accompanying wide eQTL confidence intervals does not allow distinction between trans- or cis-eQTL for genes mapped on the same linkage group, this in itself cannot explain the large differences observed.

Using the threshold for significance, we have classified genes in groups based on the amount of explained variance. Interesting to note is the reduction in the ratio between the numbers of cis- and trans-acting QTLs with increased explained variance (Table 1b). Several studies have reported a similar increase in local (cis) over trans-acting regulation, indicating that on average, cis-regulation results in stronger differential expression and thus genetic variation in comparison to trans-regulation [3,4,29-31].

**Figure 2:**
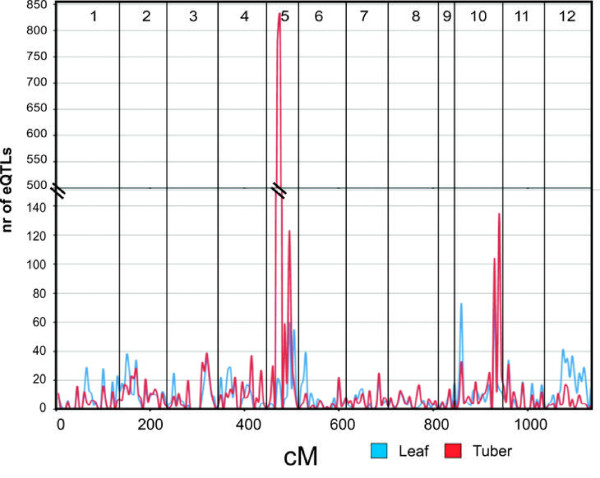
**Genome-wide trans-eQTL distribution in potato leaf and tuber**. Overview of genome-wide trans-eQTL distribution within the C × E population over the 12 potato linkage groups for tuber (red) and leaf (blue) gene expression data. The total number of significant eQTLs found within 5 cM intervals is plotted along the genetic map (cM).

One explanation for the relative high percentage of cis-eQTLs in potato could be its highly heterozygous nature and therefore possibly a tolerance to higher levels of sequence polymorphisms (SNPs and indels). Local (cis) control of transcriptional regulation may consequently be the preferred mechanism for transcriptional control in heterozygous plant species such as potato. Alternatively, the ability to detect trans-eQTLs with minor phenotypic effects is largely depending on the statistical power of the QTL study. Applying conservative filtering may result in many false negatives biasing detection towards cis-eQTLs as suggested by Petretto et al. [31]. One striking observation is the massive peak of trans-acting QTLs found on linkage group 5 (Figure 2). The question arises whether the high number of QTLs found in this small genomic region on linkage group 5 can be attributed to a single 'master switch' controlling the expression of many genes. The variation in timing of senescence and tuber formation has been phenotyped in the C × E population and has previously been mapped as the earliness locus (*El*) in the same genomic region as the eQTL hotspot found for the tuber data on chromosome 5 [32]. The C × E population shows a strong variation in the onset of tuber formation (early--late) and to allow a comparison of gene expression in tubers of equivalent physiological state, a choice was made to extract RNA of 'mature' tubers harvested several weeks after haulm killing. Two scenarios may be envisaged to explain the large number of genes of which expression is regulated by the region that harbours the earliness locus. First, the initial assumption of a similar physiological state of the sampled tubers may be false; tubers formed late in the season may still be metabolically more active, even after haulm killing, than tubers formed earlier in the season. The difference in enzymatic activities in early and late tubers could be a consequence of the observed differential gene expression levels. In this scenario, the detected eQTLs are not under the direct control of a master switch regulator but merely reflect a different metabolic status of the tubers as a result of an earlier event (timing of tuberization). An alternative explanation could be that the locus controlling plant maturity and timing of tuberization is indeed directly regulating several metabolic pathways throughout the plant life cycle. Interestingly, within the leaf expression data this hotspot was not observed. The variation in gene expression explained by the *El *locus on chromosome 5, shows that regulation of gene expression in the tuber persists even after tuber harvest and throughout storage. These observations emphasize the impact of sampling strategies on the outcome of genetical genomics experiments.

In addition to the major QTL peak on chromosome 5, further comparison of trans-acting QTL distribution between leaf and tuber data reveals few other overlapping regions of high QTL frequency in comparison to the distribution plots containing all QTLs. Overlapping trans-eQTL hotspots for both tissues can be found on LG 5 and 10 and to a lesser extent on LG 3, 9 and 11.

A total of 5,818 overlapping QTLs (spanning the same genomic region) were found in leaf and tuber, with only 401 features (6.9%) exhibiting trans-acting transcriptional control (Table 1c). It has previously been suggested that trans-acting QTLs are more often tissue specific [5,28]. In this study, we find a similar result when considering only those QTLs that were uniquely found in one of the two tissues (leaf or tuber) for which the percentages of trans-acting QTLs are elevated to 34.8% for leaf and 48.8% for tuber, respectively. Trans-acting QTLs with different eQTLs identified for the same gene in the two tissues, may be crucial in establishing organ dependent transcriptional networks. The different mRNA extraction methods used, or differences in mRNA extractability for both organs, is not likely to impact on the identification of genetic regulation of gene expression.

### Tissue dependency

Along with speciation, organ-specific gene regulation is likely to have evolved for the specific requirements of new organs or organs that have acquired new functions. The activity of different metabolic routes is organ dependent and subject to change throughout the growing season under the influence of environmental factors and developmental stage. It is therefore not surprising that tissue or developmental stage-specific eQTLs can be identified in genetical genomics studies [5,19,20]. A common approach to analyse representation of genes active in particular pathways in biological data sets is to use Gene Ontology (GO) classification (Methods). In Table 2, we compared functional classification of features with matching eQTLs in both tuber and leaf tissue against tuber and leaf specific eQTLs. In the set of matching eQTLs (present in both tuber and leaf) only few trans-acting eQTLs were found, which is consistent with the idea that trans-regulation is an important driver of differentiation (Table 1c and [5]). This is exemplified by the lack of any trans-acting eQTLs in the development- and photosystem-associated GO classes. Although most functional classes are well represented in both tuber and leaf specific eQTLs, it is interesting to note that these comprise different sets of genes, both cis- and trans-acting, which may have evolved in a tissue dependent manner. For example a considerable number of genes associated to stress response, show leaf or tuber specific QTLs likely to reflect the different environmental influences that leaves and tubers need to respond to and the differences in the responses themselves.

**Table 2:** Functional classification of cis- and trans-acting eQTLs in both leaf and tuber (overlap) or uniquely present in leaf or tuber

GO_Class	Overlap		Leaf Unique		Tuber Unique	
	***Cis***	***Trans***	***Cis***	***Trans***	***Cis***	***Trans***

**Protein**	639	30	297	170	332	411

**RNA**	361	19	174	125	260	279

**Signalling**	172	9	105	48	91	91

**Stress**	133	6	78	73	71	71

**Transport**	114	4	73	49	65	70

**Cell cycle and organisation**	101	10	45	25	58	68

**Lipid metabolism**	94	3	49	26	29	61

**Amino acid metabolism**	79	4	40	18	39	49

**Secondary metabolism**	77	2	57	33	34	41

**Hormone metabolism**	74	1	69	40	75	57

**DNA/Chromatin**	57	3	43	19	56	58

**Redox**	50	2	20	9	24	30

**Development**	45	**0**	26	14	37	33

**Cell wall**	41	3	62	24	27	53

**Nucleotide metabolism**	34	1	15	3	26	18

**Minor CHO metabolism**	33	2	15	11	15	16

**Photosystem**	32	**0**	35	19	26	**67**

**Mitochondrial/ATP synthesis**	29	3	9	12	9	11

**Major CHO metabolism**	24	1	15	7	21	15

**Tetrapyrrole synthesis**	12	2	6	4	9	**13**

**Glycolysis**	10	1	9	8	10	10

**Not assigned**	2457	289	1386	928	1414	1560

**Figure 3:**
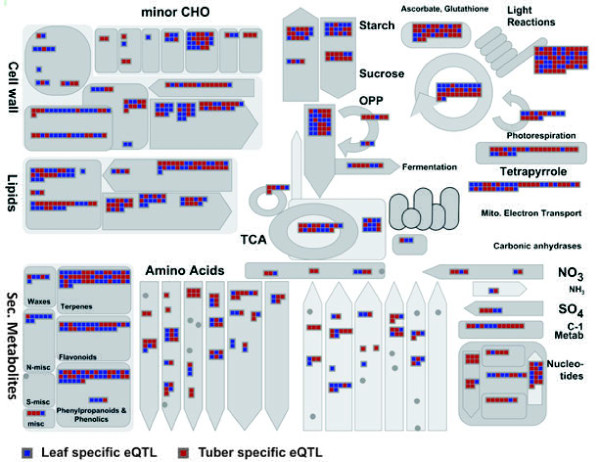
**Tissue dependent eQTLs associated to metabolic pathways**. Overview of identified tissue dependent eQTLs for genes involved in the major metabolic routes (grey boxes). Genes exhibiting potato tuber specific eQTLs are indicated in red-boxes while leaf specific eQTLs are represented in blue boxes. Each coloured box represents a single array feature and only genes for which an eQTL has been identified are visualized. Within the MapMan software additional information on gene identity and function can be obtained.

The formation of tubers requires the expression of many genes controlling the initiation of underground stems (stolons) that under favourable conditions are able to develop tubers acting as sink organs. The genome-wide RNAseq data [25] demonstrates that there are few genes that are exclusively expressed in tubers, which would imply that the tuber-specific development and response to the environment have evolved through modulation of expression and thus subfunctionalization of genes. In Figure 3, a schematic overview of important metabolic routes and associated genes is provided on the basis of presence of either tuber (red) or leaf (blue) specific eQTLs. Thus, any gene active within a metabolic pathway that is under genetic control (eQTL) is represented as either a red box (tuber) or leaf (blue box). Genes active in metabolic routes which are not under genetic regulation and thus do not produce eQTLs, are not represented. In this manner, tissue dependent variation in gene expression based on a genetic factor can be easily visualized and this provides a useful resource in the identification of key regulatory genes in the respective tissues. In both tubers and leaves, we found tissue-dependent differential expression of genes associated with major carbohydrate metabolism (CHO) (Additional file 2 and Additional file 3). In tubers, eQTLs for genes involved in the sucrose degradation and starch biosynthesis were overrepresented whereas for the leaf expression dataset, there appears to be a stronger bias towards differential regulation of starch degradation. In both tissues similar gene functions are expressed, but these are encoded by different transcripts or are differentially regulated, as reflected by the different positions of eQTLs for these genes in the two tissues. Here, we find that differentiation in the regulation of carbohydrate metabolism across tissues is strongly associated to their function, i.e. the tuber as a carbohydrate storage organ (starch synthesis) and the transient starch produced and subsequently degraded at night in the leaves. Using the MapMan visualization tool, relevant metabolic routes can be analysed in greater detail including secondary metabolites, glycolysis, transport genes or transcription factors, providing insight into tissue dependent differential regulation and whether the underlying genetic component is either cis- or trans-acting. This sort of tissue dependency not only allows the identification of genes active in the respective tissues with different mechanisms of transcriptional control (eQTLs), but also provides insight in organ dependent regulation which is the basis for tissue specific network reconstructions.

### Identification of trans-regulatory networks

**Figure 4:**
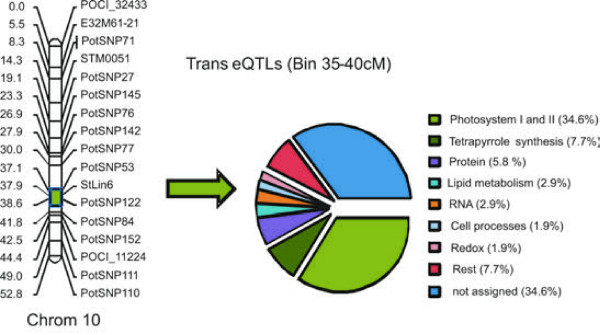
**Distribution of gene functions (GO) within a trans-eQTL hotspot on chromosome 10**. Gene Ontology (GO) classification of trans-eQTLs identified within a 10 cM region (green box) on linkage group 10 based on tuber expression data of the C × E population. Over-represented GO classes are indicated in a pie chart and percentages for each class are provided.

Co-localization of trans-acting eQTLs may indicate a similar mechanism of transcriptional control. We initially looked for over-representation of GO categories in those regions in the genome with a high number of trans-acting eQTLs from either leaf or tuber. On chromosome 10 (35-40 cM), we found a disproportionally large number of tuber-specific eQTLs (42.3%) associated with photosystem I, II, and tetrapyrrole metabolism (Figure 4 and Table 2). Greening of potato tubers after exposure to light is unacceptable for consumption and processing industry, as it is often associated with high levels of glycoalkaloid content. Edwards et al. [33], however, did not find a direct metabolic link between Chlorophyll (Chl) and glycoalkaloid biosynthesis after exposure to light. Other studies have shown genotype dependence of the tuber response to light and the ratio of glycoalkaloid and chlorophyll synthesis, as well as of physiological state and intrinsic values [34,35]. Transcriptome-wide RNAseq data show that many photosynthesis-associated genes are expressed at relatively high levels in the tuber peel [25]. During harvesting and storage, tubers may be differentially exposed to short periods of light. This may induce expression of photosynthesis-related genes, but cannot account for the genetic mapping of these expression differences. An increase in the level of expression of genes related to Chl biosynthesis may therefore reflect a heritable factor influencing sensitivity to light exposure. Interestingly, we identified functionally similar genes associated to light reactions that are differentially expressed in the leaves of the C × E genotypes (Figure 3; Light Reactions *top right corner*).

**Figure 5:**
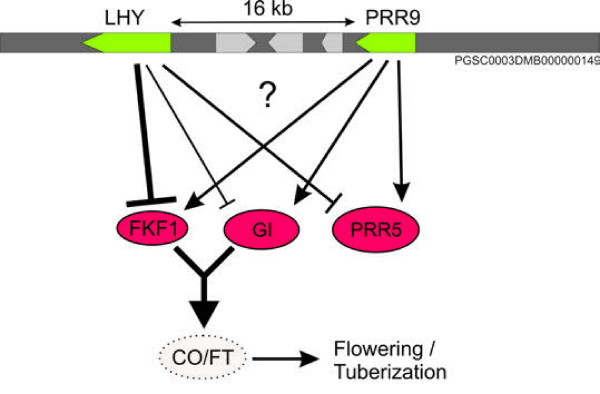
**Schematic diagram of putative gene transcriptional control mechanisms of associated circadian clock genes**. Schematic diagram of putative gene transcriptional control mechanisms of 'clock genes' based on identified cis- and trans-eQTLs on linkage group 10. Cis-eQTLs were identified for two genes part of the central clock, LHY and PRR9 (green) on scaffold PGSC0003DMB00000149. For the same genetic region, trans eQTLs were identified for genes acting downstream in clock control and part of regulatory feedback loops and are shown in red (PRR5, FKF1,GI). Variation in clock output may affect downstream genes such as constans (CO) and flowering time protein (FT) controlling development transitions. Positive or negative interactions are indicated in which the thickness of the lines represent correlation strength.

Although, over-representation of functional GO categories within eQTL hotspots can reveal regulatory networks, information on the regulation of a single or few genes can be highly relevant as well. Differential expression of genes involved in maintaining circadian rhythms and clock outputs were found to be partially regulated in the leaf by a region at the top of chromosome 10 (0-10 cM). In the genomic region spanning the QTL interval, cis-eQTLs for genes/array features with high homology to *LHY *(late elongated hypocotyl; MICRO.14662.C1) and *PRR9 *(Pseudo Response Regulator 9; PotatoF1061.scf) were identified. Both genes code for integral components of the central clock [36-39]. Interestingly, trans-acting eQTLs for genes with strong homology to members of the central clock; GIGANTEA (MICRO.7284.C1), FKF1 (MICRO.15722.C1) and Pseudo Response Regulator 5 (PRR5; MICRO.7036.C1) proteins, all mapped to the same 10 cM genomic region. The best-associated genetic marker is POCI_32433, which maps on genome scaffold PGSC0003DMB000000149 [25] and contains both the *LHY *and *PRR9 *genes, suggesting that LHY and/or PRR9 may be involved in regulation of *GIGANTEA*, *FKF1, PRR5 *genes. *LHY *expression is negatively correlated with downstream effectors *GI, FKF1 *and *PRR5*, while *PRR9 *expression is positively correlated (Figure 5). The circadian clock is involved in many aspects of plant growth and development and is associated with transition from vegetative growth to flowering and adaption to environmental and seasonal changes [reviewed in [40]]. The identification of natural genetic variation in the regulation of central clock genes may prove valuable in the unravelling of complex traits such as plant maturity, timing of flowering and yield characteristics. For potato, little is known about clock control in relation to plant growth, but timing of tuberization appears to be under direct control of the circadian clock through transcriptional regulation of *CONSTANS *(*CO*) [41] controlling the synthesis and transport of a potato flowering time ortholog associated with tuber induction [42]. In Arabidopsis, the central clock includes two MYB genes, *CCA1 *and *LHY *[37,43]. In the potato genome, only one ortholog (*LHY*) can be identified possibly indicating a different mechanism of clock control. Similar to Arabidopsis, expression patterns of *GI *and *FKF1 *in potato follow a diurnal rhythm with peak expression in the afternoon, while *LHY *expression peaks at dawn in agreement with the observed negative correlation (data not shown). In Arabidopsis, GI and FKF1 form a complex that controls CDF1 stability [44]. CDF1 in turn controls *CO *expression depending on time of day [44]. Variation in the timing of gene expression of *GI *and *FKF1 *within a population could therefore cause a phase shift or a change in phase amplitude effecting downstream effectors such as CO and FT orthologs. Here, we propose a tentative role of LHY or PPR9 in controlling the clock output by targeting expression of GI, FKF1 and PRR5 (Figure 5). Analogous to the possible difference of physiological state of the tubers at harvest as inferred from our data, the time point of sampling on the day itself may be crucial for the detection of diurnally controlled expression differences. Additional sequencing of *LHY *and *PRR9 *alleles in tetraploids can potentially reveal trait associations related to different clock control.

The strategy of deductive reasoning as used in the above examples requires a priori knowledge of the pathways and the potential interactors. For a model species such as Arabidopsis there is a wealth of scientific evidence on gene function, expression patterns and protein interactions that can be integrated in genetical genomics studies. Still, many gene functions are not yet resolved. For the majority of other plant species the annotation of biological functions of genes is often based on sequence similarity alone, which can generate various assumptions lacking scientific support. Genetical genomics studies can provide evidence supporting proposed functions of genes through newly found interactions and co-regulation of genes. This approach will lead to better understanding of transcriptional regulation, and may lead to identification of key regulatory genes underlying trait QTLs.

### Heterozygosity based QTLs (false cis-eQTLs)

Based on sequence homology of the representative unigenes to the recently published potato genome sequence, physical map positions can be attributed to a large portion of the features present on the array. However, due to the heterozygous nature of potato, the sequences of genotypes are often relatively divergent from the published genome sequence, which makes distinguishing between allelic variation, different gene family members or multi-copy genes distributed throughout the genome not straightforward. SNP frequency estimations in potato range from 1 SNP per 29 bp, found in a single phase comparison targeting 6.6 Mb of sequence [25], to 1 SNP every 87 bp between pairs of randomly selected sequences [45]. The potato unigene set used for microarray oligo design [46] brings forth similar issues, as ESTs used in the assembly originate from various tetraploid cultivars containing allelic variation. Hence, unigenes may contain allelic variation or represent different alleles of the same gene. Consequently, designed oligonucleotides based on these sequences can potentially act as allelic discriminants depending on the genotype used for array hybridizations. Hughes et al. [47] showed that even a single nucleotide mismatch can reduce signal strength up to 50% depending on the position of the mismatch. To assess allele hybridization specificity within the C × E population, we hybridized genomic DNA of both parental lines on the POCI microarray array (Methods). For 94% of the potato oligonucleotides on the array, hybridization ratios between the C and E parent could be obtained of which only 1921 features showed signal strength variation larger than 33% between both parents (Additional file 4). Of the identified cis-eQTLs, only 391 and 385 features in tuber and leaf data respectively, result in both a cis-eQTL and a differential genomic hybridization ratio (> 33%). These cis-eQTLs are likely to be false eQTLs since variation in observed signal strength may not reflect actual expression differences. Although the percentage of potential false eQTLs (4.7%) appears to be low considering SNP frequency in potato, the actual percentage may be higher due to several factors. Firstly, the C × E population is the result of a backcross, giving rise to a common allele present in both parental lines and for this common allele no specificity can be discriminated when hybridizing parental genomic DNA. Within the population however, any oligonucleotide with unique specificity for the common allele (a) will segregate in an expected 1:2:1 (aa:a-:--) ratio, resulting in differential signals that can be interpreted as expression differences. Secondly, in cases for which gene expression is relatively stable across members of the population, hybridization specificity among alleles can result in the identification of eQTLs that are based on much smaller differences than can be statistically inferred based on genomic hybridization signals. Thirdly, oligonucleotides for the array were designed based on coding sequences and when these oligonucleotides span intron-exon splicing junctions, hybridization with genomic DNA may be hindered. The use of high coverage RNAseq data would bypass most of these issues as SNP calling would allow allele distinction [48], while read counts will enable comparison of expression levels of alleles and the detection of eQTLs.

## Conclusions

In this paper we describe the identification of expression QTLs (eQTLs) in two different tissues of one of the most important food crops in the world; potato. With the completed potato genome sequence, population-wide differential gene expression can be queried to differentiate between cis- and trans-acting eQTLs. Overlapping QTLs present in both tissues are predominantly cis-acting while for tissue specific QTLs, the percentage of trans-acting QTLs increases. The type of genetic material screened and sampling strategy applied in genetical genomics studies has a high impact on the output of a genetical genomics study. Interesting regulatory networks have been identified on chromosome 10 associated to the photosystem and circadian clock control. Identification of key regulatory genes and networks unique to either source or sink tissues not only gives us insight in tissue dependent gene subfunctionalization but will also greatly enhance the identification of the causative polymorphism(s) underlying important trait QTLs.

## Methods

### Plant material

Ninety-six individuals, including the parental clones, of a diploid backcross population (C × E) were used in this study. This population is derived from an original cross between potato clones C (USW533.7) and E (77.2102.37) and is described in detail in [32]. Tuber samples originate from a field experiment, grown in repeats during the normal potato-growing season in the Netherlands (April-September). Mature tubers were collected from three plants and representative samples were mechanically peeled and immediately frozen in liquid nitrogen before being ground into a fine powder and stored at -80°C. Tuber total RNA was extracted from the 96 samples using the hot phenol method described previously [49], DNAseI treated and purified using Qiagen RNeasy columns (Qiagen). Tubers of the C × E population were planted in soil filled pots in the greenhouse and grown until stolon emergence after which fully open but still growing young leaves were collected from two replicates and immediately frozen in liquid nitrogen. Leaf RNA was isolated using KingFisher Flex system and the MagMAX™-96 total RNA isolation kit according to the manufacturer's instruction and DNAseI treated prior to labelling.

### Microarray hybridizations and data processing

All tuber samples were labelled with both Cy3 and Cy5-dye using the Low RNA Input Linear Amplification Kit, PLUS, Two-colour (Agilent technologies) according to the manufacturer's protocol starting with 2 μg of purified total RNA. Leaf samples were labelled with Cy3-dye following the same protocol as the tuber samples but starting amounts were 200 ng of purified total RNA. Hybridization and washing was performed according to the Agilent's two-colour hybridization protocol with the following change: 1 μg of labelled Cy5 and/or Cy3 cRNA was used as input in the hybridization mixture. Slides were scanned on the Agilent DNA Microarray Scanner and data extracted using the feature extraction software package (v9.1.3.1) using a standard two-colour protocol. Genes which show consistent low expression were removed and data sets were independently normalized using the quantile normalization procedure (mean) available in Genstat^® ^11.1. As tuber samples were measured twice, only genes with a Pearson correlation coefficient higher than 0.8 across the 94 individuals between the Cy3 and Cy5 datasets were included resulting in 19,956 features. For calculation of genome distribution in tubers the Cy3 data was used. All raw and normalized data files are available as Additional files 5, 6, 7, 8, 9, 10, 11, 12, 13,&14 including annotation and genome mapping information. All normalized expression data for leaf and tuber samples have been deposited in ArrayExpress (E-MTAB-808, E-MTAB-701).

Genomic DNA hybridizations were performed after labelling 800 ng of genomic DNA from both parents (C and E) in duplo, using the BioPrime^® ^labelling kit (Invitrogen) with a modified dNTPmix (1.2 mM of dATP, dGTP, dTTP, 0,6 mM dCTP and 5 ul dCTP-Cy3 or dCTP-cy5 Agilent technologies) and incubated for 16 h at 37°. Labelled samples were purified using PureLink™ PCR purification System (Invitrogen) and heat fragmented for 30 min resulting in fragments ranging from ~150 to 600 bp. 1 ug of labelled samples (cy3 and cy5) were hybridized at 65° for 17 h in a standard swop-dye experiment using the independently labelled samples. Washing and scanning of the slides was carried out as described for the gene expression experiments. Feature normalization and ratio calculations were carried out using default method available in Agilent Feature extraction software package (v9.1.3.1). Features showing consistent differential expression (> 33%) between both parents are presented in Additional file 4.

### Genetic map and linkage to the potato genome

The genetic map used in all QTL studies was generated using mapping software Joinmap 4.0^® ^[50] and is a modified version of an earlier C × E genetic map [51], including all sequence based SNP markers. Additional markers were obtained from the tuber expression data set, in cases where both cy3 and cy5 hybridization signals could be unambiguously scored as present or absent. Marker names originating from tuber expression data have POCI as a prefix. All sequence based markers present in the map are linked to EST contigs or EST singletons and these sequences were blasted against the potato genome scaffolds (v3.4). Segregating markers with unique and significant genome scaffold hits (> 90% homology) could be subsequently linked to the physical genome map (Additional file 15). The C × E generated genetic map was validated aligning the sequence based markers along the draft scaffold/pseudo-molecules available from the PGSC website [25]. The potato oligo (60-mer) microarray (POCI) used in the experiments, contains 42,034 features based on a potato unigene set [46]. To allow discrimination between cis and trans-eQTLs all unigenes were blasted against the genome scaffolds sequences, predicted Coding sequences (CDS) and predicted gene regions (including 5' and 3'-UTR's). Features with a unique and significant hit were assigned to genome scaffolds for which the majority has chromosome information [25] and results are presented in Additional file 16. Identified QTLs on the same linkage group as their physical map position are identified as cis-acting while QTLs on different linkage groups are defined as trans-acting. Features on the array for which no physical map position could be assigned are classified as unknown.

### QTL analysis

We have used the main module of the R/qtl package, and optimized it for high-throughput QTL mapping in outcrossing species such as potato. The R-script checks the ratio of missing values before automatically converting JoinMap segregation scores to R/qtl scores after which the QTL mapping is performed [52]. The genotypic scores and genetic map order used in this study are available in Additional file 15 and 17. The program relies on R/qtl for the QTL mapping where QTL information is extracted from the summary.scanone method and the explained variance of the QTL is computed for each QTL using the *makeqtl *and the *fitqtl *functions. The QTL interval is computed using LOD interval method. The default analysis performs a QTL mapping using the Haley-Knott regression with a mapping step size of 5 cM. The LOD threshold used to determine the QTLs is calculated using Li & Ji algorithm [53]. The number of simulation replicates to perform for the sim.geno function is default to 16 and the n.draws parameter allows further adjustment. For tuber and leaf expression data QTL analysis was run using the LOD interval method with default step size and significance LOD threshold of 4.35.

### Gene ontology and eQTL visualization

Gene ontology (GO) assignment for all the potato micro-array features has been described previously (Kloosterman et al. 2008). GO information was downloaded through Agilent E-array published designs (https://earray.chem.agilent.com/earray/). The number of array features having eQTLs (cis or trans) were counted per GO group and totals are listed. To reduce the number of classes, GO identifiers targeting the same metabolic routes were in some cases merged (Table 2). GO classification of the POCI microarray has been previously linked to the expression data visualization tool MapMan (Kloosterman et al. 2008). MapMan is a user-driven tool that normally displays large datasets (e.g. gene expression data arrays) onto diagrams of metabolic pathways or other processes (http://mapman.gabipd.org/web/guest/mapman). Instead of visualizing gene expression data we display the presence of tissue specific eQTLs (eQTL present in leaf = blue box; eQTL present in tuber = red box). In this manner a quick overview can be obtained for each pre-designed metabolic route and genes can be identified that are differentially expressed within a segregating population (Figure 3). Additional information on gene name and function can be obtained for each box when running the MapMan software (publicly available).

## Abbreviations

SNP: Single nucleotide polymorphism; QTL: Quantitative trait loci; LOD: Logarithm of odds

## Authors' contributions

BK designed the tuber expression study, performed QTL analysis, data interpretation and wrote the manuscript. AMA designed and performed the leaf expression study and carried out hybridizations together with MO. PC optimized the R/qtl program and performed QTL and statistical analysis. GL designed the leaf expression study and helped draft the manuscript. RGFV and CWBB helped design the tuber expression experiment and help draft the manuscript. All authors read and approved the final manuscript.

## Supplementary Material

Additional file 1 **Frequency distribution of eQTLs over the different potato chromosomes**. Click here for file

Additional file 2 **Tissue specific and Tissue dependent eQTLs of tuber and leaf tissues belonging to the major carbohydrate metabolism groups**. Click here for file

Additional file 3 **Schematic overview of tuber and leaf eQTL specificity for major CHO metabolism genes**. Click here for file

Additional file 4 **Hybridization specificity of genomic DNA from C and E parental clones**. Click here for file

Additional file 5 **Tuber Cy3 and Cy5 raw data + genome info partI**. Click here for file

Additional file 6 **Tuber Cy3 and Cy5 raw data + genome info partII**. Click here for file

Additional file 7 **Tuber Cy3 and Cy5 raw data + genome info partIII**. Click here for file

Additional file 8 **Tuber Cy3 and Cy5 qnormalized data + genome info partI**. Click here for file

Additional file 9 **Tuber Cy3 and Cy5 qnormalized data + genome info partII**. Click here for file

Additional file 10 **Tuber Cy3 and Cy5 qnormalized data + genome info partIII**. Click here for file

Additional file 11 **Leaf Cy3 Leaf raw data + genome info partI**. Click here for file

Additional file 12 **Leaf Cy3 Leaf raw data + genome info partII**. Click here for file

Additional file 13 **Leaf Cy3 qnormalized + genome info partI**. Click here for file

Additional file 14 **Leaf Cy3 qnormalized + genome info partII**. Click here for file

Additional file 15 **Marker to Genome**. Click here for file

Additional file 16 **4x44k 015425 POCI annotation**. Click here for file

Additional file 17 **Genotypic scores CxE population**. Click here for file
