# Supplementary material for: Organ specificity and transcriptional control of metabolic routes revealed by expression QTL profiling of source--sink tissues in a segregating potato population
Source: BMC Plant Biol. 2012 Feb 7;12:17. doi: 10.1186/1471-2229-12-17 (PMC3546430; doi:10.1186/1471-2229-12-17)
Supplement: Additional file 3 — Schematic overview of tuber and leaf eQTL specificity for major CHO metabolism genes. [file 1471-2229-12-17-S3.PDF]

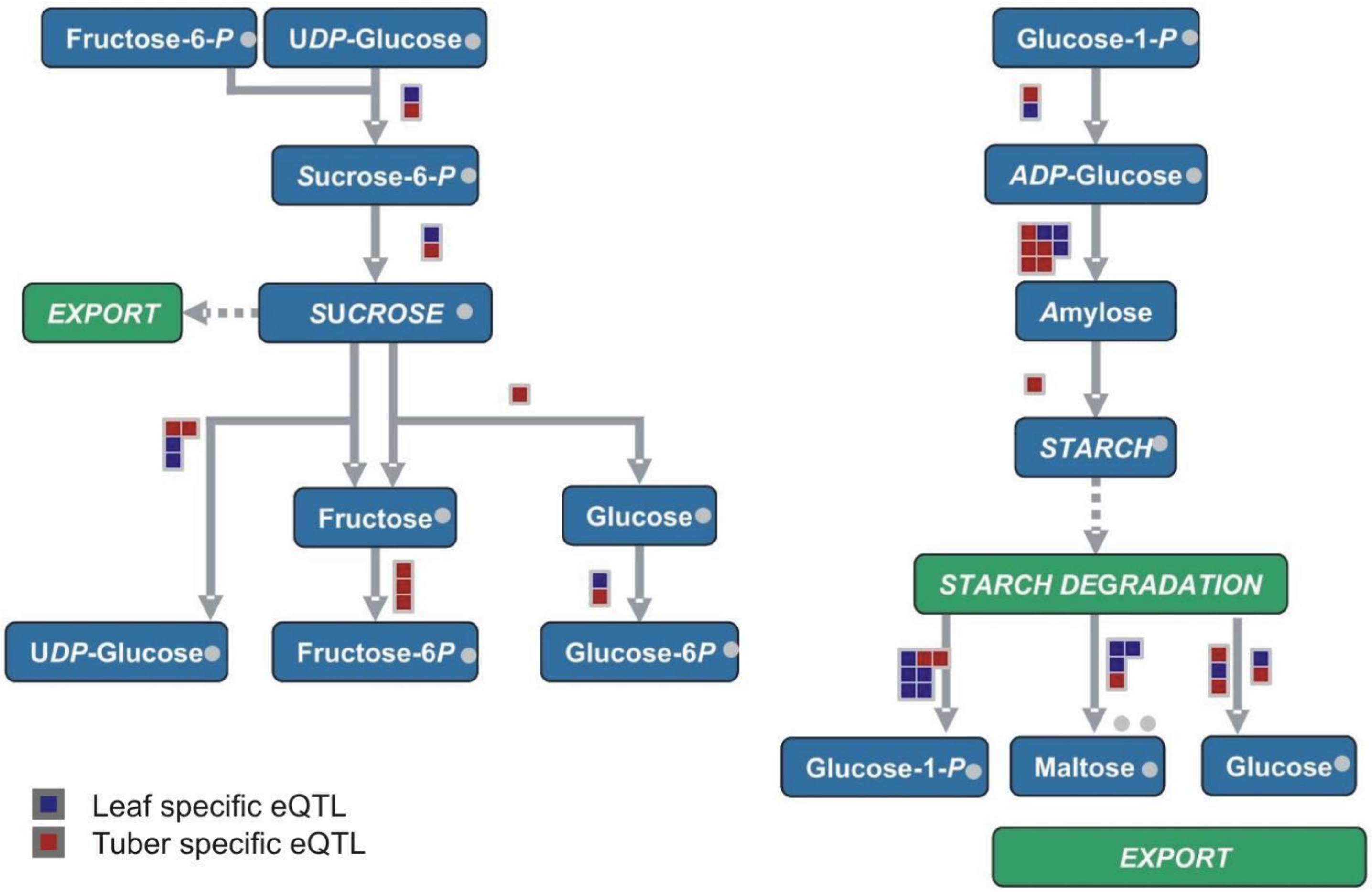

Additional file 3: Tissue dependent eQTLs for genes involved in part of the major carbohydrate (CHO) routes with tuber specific eQTLs in red and leaf specific eQTLs indicated in blue boxes. Each coloured box represents single array feature.
